# Transcriptome profiling of *Bergenia purpurascens* under cold stress

**DOI:** 10.1186/s12864-023-09850-z

**Published:** 2023-12-07

**Authors:** Xuebin Zhang, Fang Yu, Xin Lyu, Jingyu Chen, Hongyan Zeng, Nuomei Xu, Yufeng Wu, Qiankun Zhu

**Affiliations:** Sichuan Engineering Research Center for Biomimetic Synthesis of Natural Drug, School of Life Science and Engineering, Southwest Jiaotong University, Chengdu, 610031 China

**Keywords:** *Bergenia purpurascens*, RNA-seq, Transcriptome, Cold stress

## Abstract

*Bergenia purpurascens* is an important medicinal, edible and ornamental plant. It generally grows in high-altitude areas with complex climates. There have been no reports about how *B. purpurascens* survives under cold stress. Here, the *B. purpurascens* under low temperature were subjected to transcriptomics analysis to explore the candidate genes and pathways that involved in the cold tolerance of *B. purpurascens*. Compared with the control treatment, we found 9,600 up-regulated differentially expressed genes (DEGs) and 7,055 down-regulated DEGs. A significant number of DEGs were involved in the Ca^2+^ signaling pathway, mitogen-activated protein kinase (MAPK) cascade, plant hormone signaling pathway, and lipid metabolism. A total of 400 transcription factors were found to respond to cold stress, most of which belonged to the MYB and AP2/ERF families. Five novel genes were found to be potential candidate genes involved in the cold tolerance of *B. purpurascens*. The study provide insights into further investigation of the molecular mechanism of how *B. purpurascens* survives under cold stress.

**Supplementary Information:**

The online version contains supplementary material available at 10.1186/s12864-023-09850-z.

## Introduction

*Bergenia purpurascens* (Hook.f.et Thoms.) Engl., also called *Purple Bergenia*, is an important medicinal, edible, and ornamental plant belonging to the genus *Bergenia* in the *Saxifragaceae* family. It grows extremely slowly and is mainly distributed in highland areas of southwest China and other Asian regions [1]. The whole grass and rhizome can be used as medicine. *B. purpurascens* is rich in active polyphenolic components like bergenin and arbutin. Bergenin has been successfully developed as a listing drug (Compound Bergenin Tablets) for treating chronic bronchitis [2]. It also treats other diseases such as pneumonia, tuberculosis, gastric ulcer, and acute [3]. At the same time, it has food and ornamental value. *B. purpurascens* not only has diverse functions but also has strong ecological adaptability. It grows at high altitudes of 2800–4800 m, where the temperature is characterized by a significant difference between day and night and a low average annual temperature [1]. Due to constant exposure to cold stress, *B. purpurascens* has evolved to tolerate cold stress and is likely to be more winter-hardy than other species of *Bergenia* [4]. It is a potential ideal material for the study of cold resistance in plants.

Several recent studies have explored the adaptation mechanism of plants grown in high-altitude areas to cold stress. For example, physiological and transcriptome analyses were performed for *Ammopiptanthus mongolicus* surviving in the Qinghai-Tibetan Plateau after freezing stress (-7.5 °C) [5]. He et al. [6] compared the cold resistance physiological and biochemical features of four *Herba Rhodiola* seedlings (grown at altitude over 3400 m) under low temperature. Metabolic analyses were conducted on Tibetan hulless barley (*Hordeum distichon*) exposed to six temperatures (24 °C, 12 °C, 5 °C, 0 °C, -5 °C, -8 °C) [7]. To date, research on *B. purpurascens* mainly focused on its pharmacological value and chemical components, as well as its morphology and growth characteristics [1, 8]. Few research on molecular biology or its genomic data, not to mention the cold resistance mechanism for *B. purpurascens*.

Low temperature (cold stress) affects plants’ geographical distribution, leading to reduced yields and threatening plant survival [9]. Cold stress can be classified as chilling stress (0 to 20 °C) and freezing stress (< 0 °C) [10]. Cell membrane structure and fluidity would change under low temperature condition [11]. The cell membrane changes can be sensed by calcium channels and proteins localized on the plasma membrane, leading to the changes in Ca^2+^ flow and the regulation of low-temperature responsive genes [12]. The CBF (C-repeat binding factors) pathway is one of the most widely reported pathways that responds to low temperature [13]. In addition, plant hormones are essential for plant cold acclimation, including abscisic acid (ABA), gibberellin (GA), ethylene, brassinosteroids (BR) and Jasmonic acid(JA) [14].

Currently, high-throughput sequencing technologies have been widely used in the study of the mechanism of plant response to biotic or abiotic stress [15]. The transcriptomic level of plants generally changes over a short period in response to stress [16]. To explore the mechanisms of *B. purpurascens* adaptation to cold stress, we performed transcriptome sequencing on *B. purpurascens* leaves treated at 4 °C for 6 h. Our study provides gene information resources for the analysis of the cold resistance mechanism of *B. purpurascens*, as well as a reference for the breeding of resistant species of plants.

## Materials and methods

### Plant growth and cold treatment

The *B. purpurascens* seedlings for this study were collected on October 13, 2022 in Jiulong County, Sichuan Province, China (~ 101.50°N, ~ 29.00°E), and planted into a mixture of peat soil and gravel at 22 °C under long-day conditions (16 h light of 70 µE m^− 2^ s^− 1^ and 8 h dark). The altitude of the sample collection site is 2900 m, where the annual average low temperature was 4 °C and the annual average high temperature was 18 °C in 2021 (meteorological data source: website: https://www.tianqi24.com/jiulong/history2021. html). The species information was confirmed by DNA barcoding technique with *ITS2* and *psbA-trnH* sequences [17]. After being incubated at 4 and 22 °C for 6 h under light of 70 µE m^− 2^ s^− 1^, respectively, the first completely expanded leaves from the top were transferred into sample tubes followed by being immediately frozen in liquid nitrogen and finally stored at -80 °C. The 6 h time-point and 4 °C were selected based on the growing conditions of *B. purpurascens* and previous studies about cold stress on *Sonneratia caseolaris*, *Populus tomentosa*, *Oxytropis ochrocephala* and *Arabidopsis* [16, 18–20]. After these plants were transferred to 4 °C for 6 h, the transcriptome level changed dramatically.

### cDNA library preparation and sequencing

The total RNA of the first completely spread leaves from the top was extracted using Trelief® RNAprep Pure Plant Kit (Tsingke, China). RNA concentration and purity were examined using Nanodrop 2000 (Thermo Fisher Scientific, USA), RNA integrity was examined through agarose gel electrophoresis, and RIN values were determined by Agilent 5300 Bioanalyzer (Agilent, USA). After the RNA samples reached the qualified level, the library assembly and RNA-seq analysis were performed on the Illumina Novaseq 6000 high-throughput sequencing platform with the assistance of Shanghai Meiji Biotechnology Company (Shanghai, China). Three biological replicates were performed.

### De novo transcriptome assembly

To ensure the quality of the transcriptome sequence results and bioinformatics analysis, the raw sequences (raw reads) obtained from the sequencing were counted and quality controlled by fastp (v.0.19.5, https://github.com/OpenGene/fastp). Then Trinity (v.2.8.5, https://github.com/trinityrnaseq/trinityrnaseq) was used to splice the filtered clean reads to obtain the reference sequences for subsequent bioinformatics analysis. Gene function annotation was performed using six major databases: NR (NCBI non-redundant protein Sequences), Swiss-Prot (A manually annotated and reviewed protein sequence database), GO (Gene Ontology), KEGG (Kyoto Encyclopedia of Genes and Genomes), eggNOG (Evolutionary Genealogy of Genes: Non-supervised Orthologous Groups) and Pfam (Protein family), with E-value ≤ 1e^− 5^.

### Differential expression analysis and functional enrichment

Gene expression levels were calculated by RSEM (v1.3.1, http://deweylab.biostat.wisc.edu/rsem/) [21], and the expression level of the unigenes was converted to TPM (transcripts per million) value. Differential expression analysis was performed by DESeq2 (v.1.24.0, http://bioconductor.org/packages/stats/bioc/DESeq2/). Differentially expressed genes between samples were screened with log_2_|Fold Change| ≥ 1&Padjust < 0.05. The Python-based library GOATools was used for GO enrichment. The KEGG database was used for enrichment analysis of the signaling pathways of the differentially expressed genes to screen out the functional genes associated with cold stress [22].

### Quantitative real-time PCR (qRT-PCR) validation

RNA was extracted from cold-treated and control-treated samples. First-strand cDNA was reverse transcribed from RNA using Evo *M-MLV* RT mix Kit with gDNA Clean for qRT-PCR Ver.2 (Accurate Biology, China). Ten genes (*WRKY33*, *ZAT10*, and eight randomly selected genes) were performed on qRT-PCR to validate the RNA-seq data. 18 S rRNA was used as the internal reference to normalize the expression data [23]. qRT-PCR was performed with SYBR GREEN I (Accurate Biology, China) on the Analytik Jena qTOWER^3^G fluorescent quantitative PCR instrument (Analytik Jena AG, Germany). The cycling conditions were 95 °C pre-denaturation for 30 s, 95 °C for 5 s, 60 °C for 30 s, 40 cycles. Primers used for qRT-PCR were designed by Primer-Blast and shown in Table S1. Three biological replicates were generated. The 2^−ΔΔCt^ method was used to calculate the relative gene expression. RNA-seq value was based on the fold change of up-regulated or down-regulated DEGs.

## Results

### Transcriptome sequencing and de novo transcriptome assembly

RNA libraries were constructed and sequenced for a total of six samples from two groups of *B. purpurascens* samples, including control (CK) and cold (4 °C) treatment. Among them, there were 297,586,224 original fragments. After removing low-quality areas and adapters, 285,734,142 clean reads remained. Q30 (sequences with a sequencing error rate of less than 0.1%) were all above 93%, and the average GC content was 44.97% (Table 1). Clean data from 6 samples were mapped to the assembled unigenes using Trinity. The result showed that the number of unigenes obtained was 109,971.

**Table 1:** Assembly quality statistics

Sample	Raw reads	Raw bases	Clean reads	Clean bases	Q30 (%)	GC content (%)
CK_1	48,639,092	7,344,502,892	46,945,854	6,874,791,843	93.71	45.06
CK_2	48,016,470	7,250,486,970	47,033,282	6,836,442,585	94.40	45.05
CK_3	57,535,484	8,687,858,084	53,728,440	7,835,216,183	93.13	45.30
Cold_1	47,733,804	7,207,804,404	46,555,808	6,810,153,024	94.04	44.70
Cold_2	46,632,624	7,041,526,224	45,616,444	6,605,959,084	94.43	44.86
Cold_3	49,028,750	7,403,341,250	45,854,314	6,730,151,950	93.05	44.84
total	297,586,224	44,935,519,824	285,734,142	41,692,714,669	—	—

### Functional annotation and DEGs screening

**Fig. 1:**
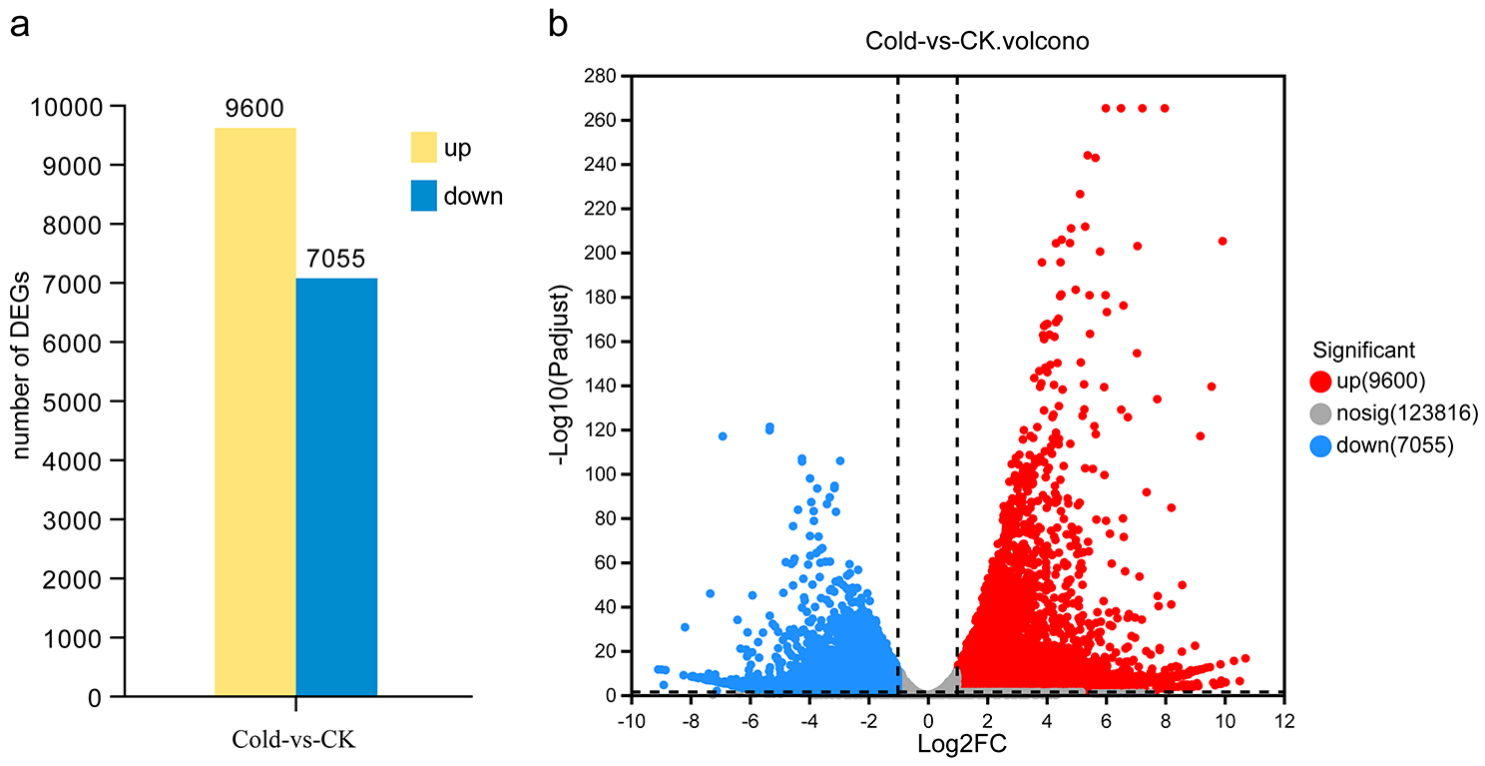
Differentially expressed genes (DEGs) in *B. purpurascens* in response to cold stress compared to control (CK). (**a**) Column diagram. (**b**) Volcano plot. The genes meeting Padjust < 0.05 & |log2FC| ≥ 1 were considered as differentially expressed genes

The obtained unigenes were subjected to NR, Swiss-prot, GO, KEGG, eggNOG, and Pfam public databases to obtain annotation information. 48,668 unigenes were annotated in at least one database within a predefined range of E-value. There were 47,573 (43.26%), 30,647 (27.87%), 39,987 (36.36%), 20,134 (18.31%), 33,838 (30.77%) and 24,055 (21.87%) unigenes annotated in NR, Swiss-prot, GO, KEGG, eggNOG, and Pfam, respectively (Table 2). Based on the read counts, DEGs were assessed with the DESeq2 program. According to Padjust < 0.05 and |log2FC| ≥ 1, a total of 16,655 differentially expressed genes (DEGs) were obtained after cold stress treatment, of which 9,600 were up-regulated and 7,055 were down-regulated (Fig. 1, Table S2).

Besides screening the homologues of the previously reported genes, transcriptomic data also have the potential to discover novel genes. Based on the fold changes of expression, we selected the genes with high expression level (average TPM > 50 in both control and cold-treated groups). Among the ten up-regulated and ten down-regulated genes with the most significant changes in expression, five genes (DN12077_c0_g1, DN12077_c1_g1, DN5468_c1_g1, DN7548_c0_g1, DN95039_c0_g1) were unknown and failed to be annotated (Table S3). These genes were proposed as potential novel candidate genes implicated in the cold tolerance of *B. purpurascens*.

**Table 2:** Statistics of functional annotation of non-redundant unigenes sequenced for *B. purpurascens* against public databases

	Number of unigene	Percentage
Annotated in NR	47,573	43.26%
Annotated in Swiss-Prot	30,647	27.87%
Annotated in GO	39,987	36.36%
Annotated in KEGG	20,134	18.31%
Annotated in eggNOG	33,838	30.77%
Annotated in Pfam	24,055	21.87%
Annotated in at least one Database	48,668	44.26%
Annotated in all database	7,352	6.69%
Total unigenes	109,971	1

### GO and KEGG enrichment under cold stress

**Fig. 2:**
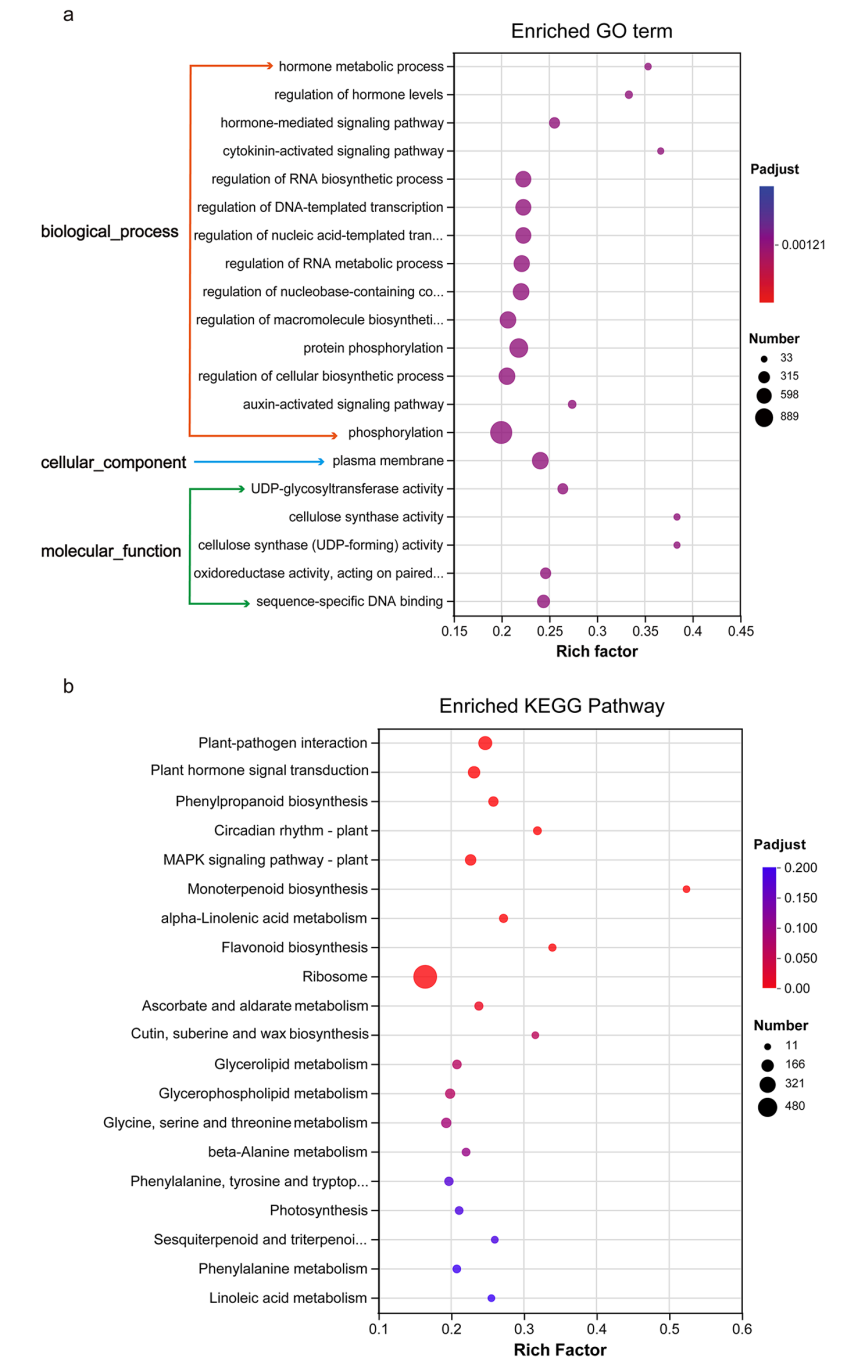
GO and KEGG enrichment analysis of differentially expressed genes (DEGs) in response to cold stress compared to control. (**a**) GO enrichment of top 20 terms. (**b**) KEGG enrichment of top 20 pathways

To uncover the function of DEGs, GO enrichment and KEGG enrichment were performed. For GO enrichment, GO terms were classified into three groups: cellular component, biological process, and molecular function. Among the top 20 significantly enriched GO terms (Fig. 2a), 14 were related to biological process, one to cellular component, and five to molecular function. In the biological process, hormone metabolic processes (GO: 0042445), hormone level regulation (GO: 0010817), and hormone-mediated signaling pathways (GO: 0009755) were the most significantly enriched GO terms. KEGG enrichment results showed that 2,093 DEGs under cold stress were enriched into 140 metabolic pathways. As shown in the top 20 items of KEGG enrichment analysis (Fig. 2b), plant-pathogen interaction (Ko04626), plant hormone signal transduction (Ko04075), phenylpropanoid biosynthesis (Ko00940), circadian rhythm-plant (Ko04712), and MAPK signaling pathway (Ko04016) were significantly enriched. In addition, five of the top 20 items were related to lipid metabolism, including alpha- Linolenic acid metabolism (Ko00592), cutin, suberine and wax biosynthesis (Ko00073), glycerolipid metabolism (Ko00561), glycerophospholipid metabolism (Ko00564), and linoleic acid metabolism (Ko00591).

### Ca^2+^ signal transduction

**Fig. 3:**
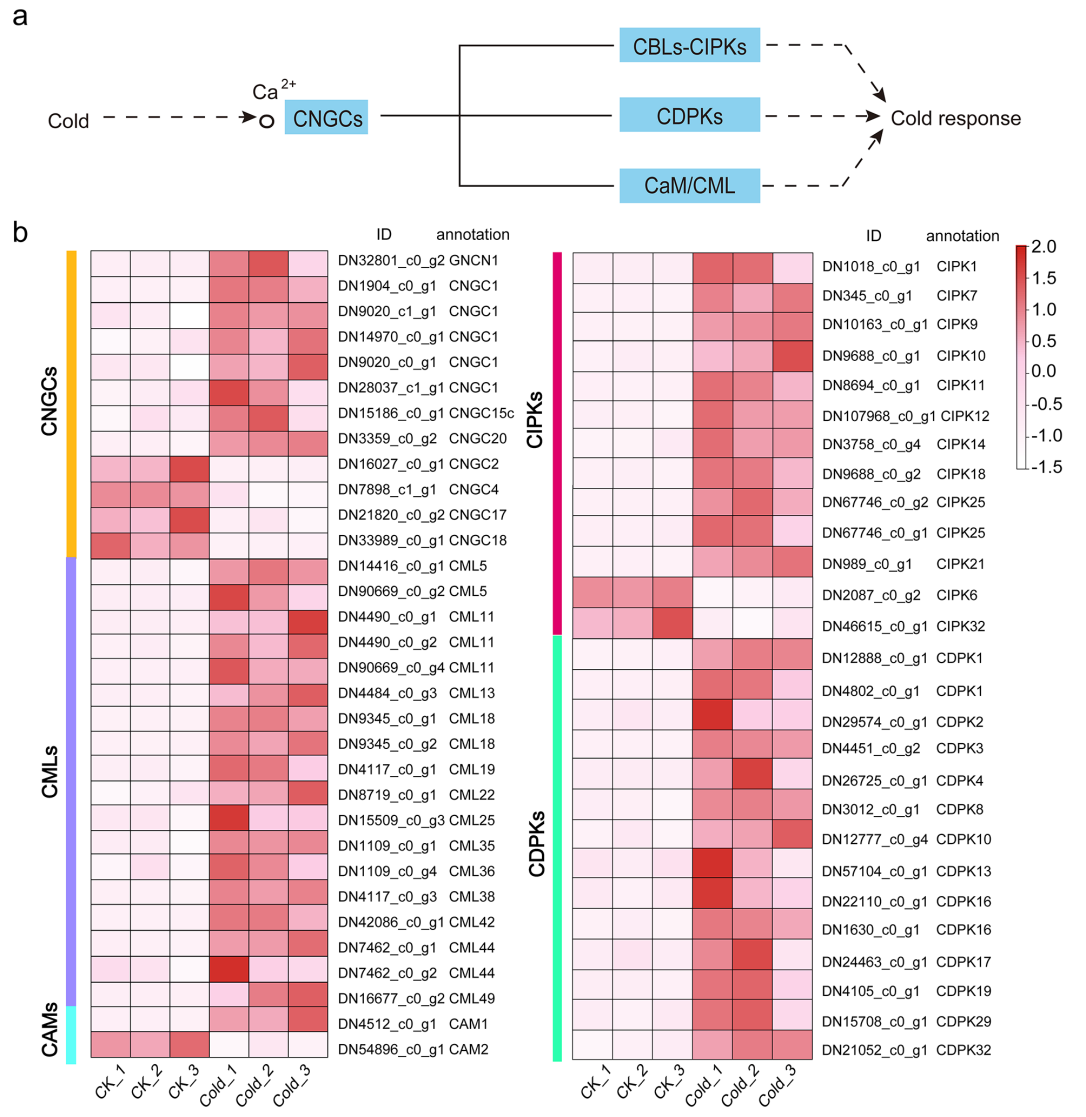
Ca^2+^ signal in response to cold stress. (**a**) Ca^2+^ signaling pathway schematic display. (**b**) Heatmap of DEGs involved in Ca^2+^ signaling pathway

The calcium ion (Ca^2+^) is an important secondary signaling molecule and plays a vital role in the cold stress response of plants [24]. Figure 3 and Table S4 showed the DEGs identified to be related to Ca^2+^ signal transduction under cold stress in *B. purpurascens*. Among them, there were 51 DEGs up-regulated and seven down-regulated. CNGCs are non-selective cation channels that can serve as a pathway for Ca^2+^ conduction into the cytoplasm [25]. In our study, among 12 *CNGC*-homologous DEGs, eight were up-regulated (Fig. 3b). Ca^2+^ sensors include calmodulins (CaMs), CaM-like proteins (CMLs), calcineurin B-like proteins (CBLs), Ca^2+^-dependent protein kinases (CPKs/CDPKs), and calcineurin B-like-interacting protein kinases (CIPKs) regulated by CBLs [26]. As shown in Fig. 3b, we found 46 DEGs related to Ca^2+^ sensors, including two *CaMs*, 17 *CMLs*, 14 *CDPKs*, and 13 *CIPKs*.

### MAPK signal transduction

**Fig. 4:**
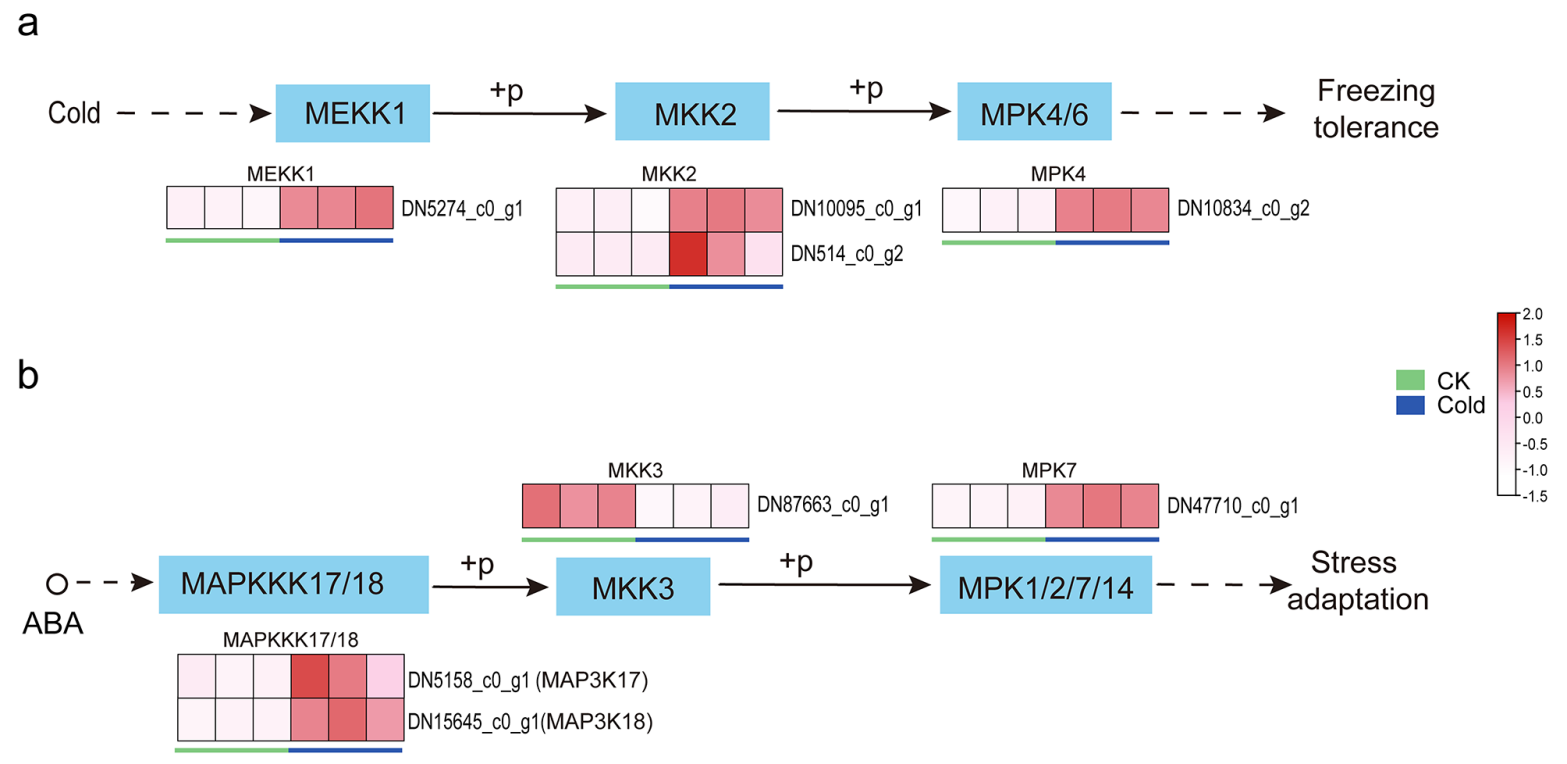
MAPK signal in response to cold stress. (**a**) MEKK1-MKK2-MPK4/6 pathway induced under cold stress. (**b**) MAPK signaling pathway induced by ABA in response to cold stress

In this study, the genes related to MAPK signal transduction led to significant change under cold stress (Table S5). The expression levels of the pathway genes *MEKK1* (DN5274_c0_g1), *MKK2* (DN10095_c0_g1, DN514_c0_g2), and *MAPK4* (DN10834_c0_g2) were significantly increased when the samples were exposed to cold stress (Fig. 4a). In the MAPK signaling pathway activated by ABA, *MAPKK17* / 18 (DN15645_c0_g1, DN5158_c0_g1) and *MPK7* (DN47710_c0_g1) were up-regulated (Fig. 4b).

### Plant hormone signal transduction

**Fig. 5:**
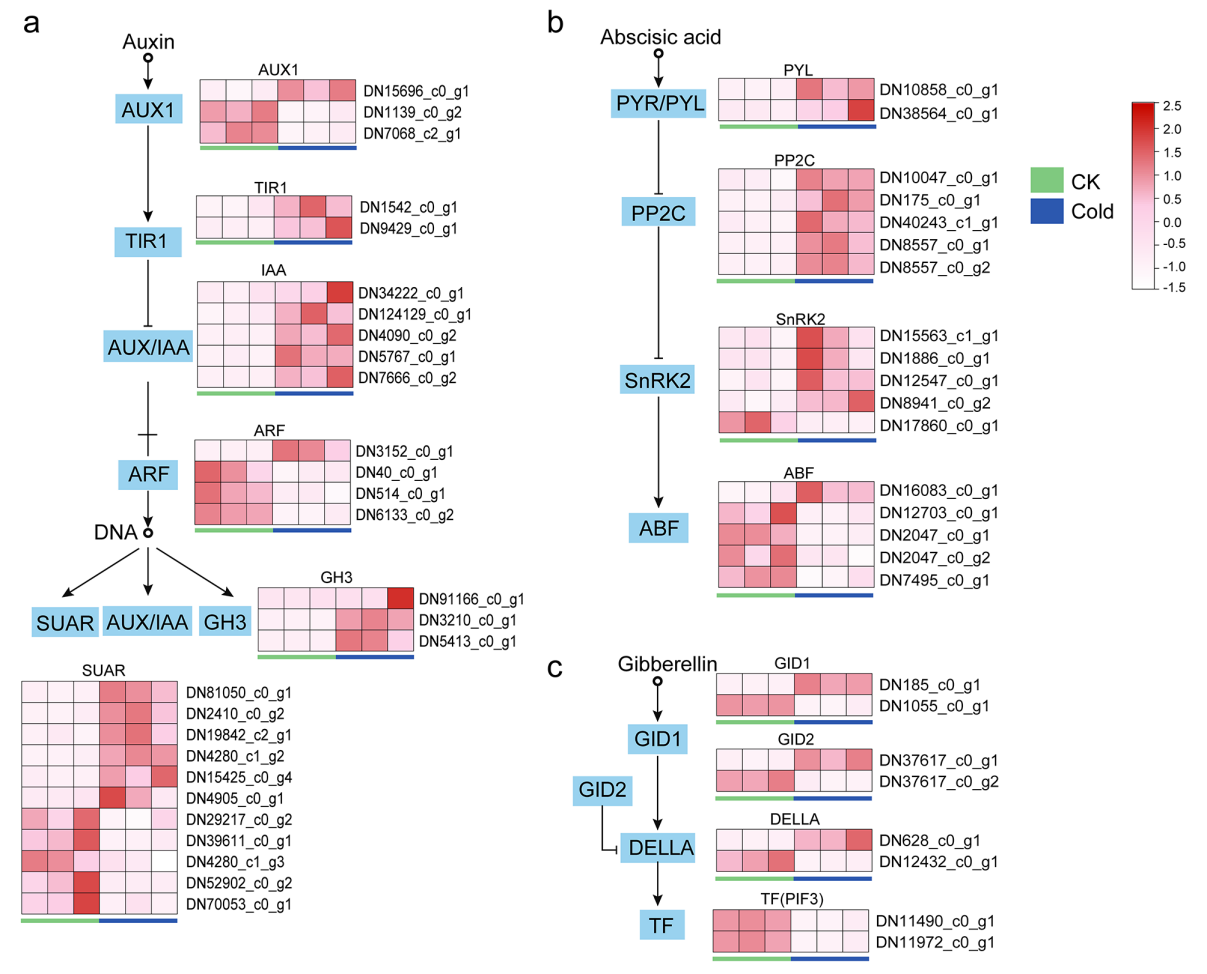
Plant hormone signal in response to cold stress. (**a**) Auxin, (**b**) Abscisic acid, (**c**) Gibberellin

Plant hormones play an important role in the regulation of plant growth and response to abiotic stress [27]. Under cold conditions, environmental stress factors induce the accumulation of plant hormones and trigger the plant hormone signaling pathway [14]. In our study, the results of KEGG enrichment showed a significant amount of DEGs enriched in plant hormone signal transduction (Table S6). Figure 5 showed DEGs in auxin, abscisic acid (ABA), and gibberellin (GA) signal transduction [28–30]. Additionally, Figure S3 showed part of the ABA synthesis pathway. In *B. purpurascens*, multiple homologues of *ARF* in auxin, *ABF* in ABA, and *PIF3* in GA were down-regulated under cold stress. Moreover, multiple homologues of *TIR1*, *IAA* and *GH3* in auxin, and *PYR/PYL*, *PP2C* and *SnRK2* in ABA were up-regulated.

### Transcription factors responding to cold stress

**Fig. 6:**
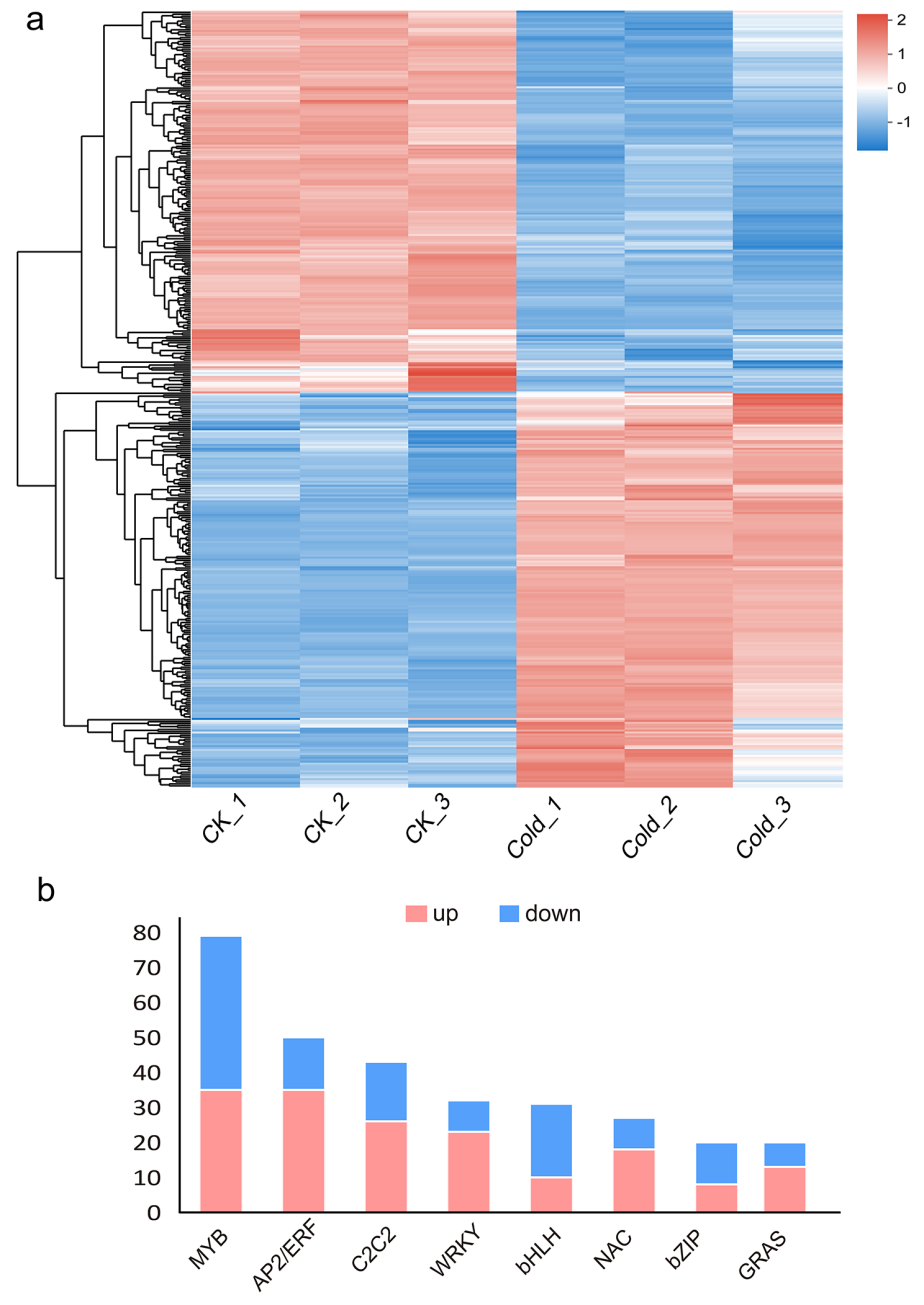
Differentially expressed transcription factors in *B. purpurascens* under cold stress. (**a**) Hierarchical clustering of 400 differentially expressed transcription factors. Expression values (TPM) are log2 - transformed. The colors indicate the gene expression levels from low (blue) to high (red). The detailed information with unigene IDs and annotations is shown in Table S6. (**b**) The distribution of the top 8 differentially expressed transcription factors in up-regulated and down-regulated categories

In our study, we predicted 1,114 transcription factor genes from the *B. purpurascens* transcriptome. Among them, 400 transcription factors distributed across 26 gene families were identified as DEGs in response to cold stress, including 79 MYB transcription factors, 50 AP2/ERF transcription factors, 42 C2C2 transcription factors, 32 WRKY transcription factors, 31 bHLH transcription factors, and 27 NAC transcription factors (Table S7, Fig. 6). Based on the result of the hierarchical clustering (Fig. 6a), 400 transcription factors were divided into down-regulation group and up-regulation group with similar numbers. The top three up-regulated families were MYB, AP2/ERF and C2C2, and the top three down-regulated were MYB, C2C2 and bHLH (Fig. 6b). In addition, several well-known cold-related transcription factors, including *CBF1* belonging to AP2 gene family and *MYB15*, were differentially expressed under cold stress as shown in Figure S1.

### Validation of transcriptome data by qRT-PCR analyses

To confirm the DEGs obtained from Illumina Novaseq 6000 platform sequencing were credible, the qRT-PCR was performed on RNA extracted from cold and control-treated samples. Ten DEGs were selected for qRT-PCR, including two transcription factors (*WRKY33* and *ZAT10*) and eight randomly selected genes (Figure S2). Comparison of qRT-PCR and RNA-seq showed that although there were small differences in the expression variability of these genes, the expression trends were consistent. The results suggested that the transcriptome data were reliable.

## Discussion

Few studies have reported the genome and transcriptome of *B. purpurascens*, which hinder the understanding of the molecular mechanisms of its adaptation to severe highland environments and tolerance to abiotic stress. To our knowledge, this is the first transcriptomic analysis of *B. purpurascens* in response to cold stress. The discovery of cold-response genes and pathways in the study provided valuable insights into underlying the molecular mechanisms of cold tolerance of *B. purpurascens*. The study showed that a significant number of cold-response genes were involved in diverse biological processes, primarily encompassing lipid metabolism, Ca^2+^ signaling, MAPK, plant hormones, and transcription factors, which require further discussion.

### DEGs involved in lipid metabolism

When the temperature decreases, the cellular membrane functions as the primary location for sensing temperature signals [9]. Lipids are one of the major components of biological membranes [31]. In the present study, KEGG enrichment suggested that numerous DEGs were significantly enriched in lipid metabolism pathways (Fig. 2b). These pathways might be related to the changes in membrane lipid composition in *B. purpurascens* under cold stress, which was similar to the membrane lipid change-related pathways in blueberry in response to cold stress [32]. Szymanski et al. [33] elucidated the association between the expression of lipid metabolism genes and membrane lipid composition. Studies conducted on potato [34] and pineapple [35] also showed that temperature alterations could induce changes in lipid components in the cell membrane to reduce cold damage. Meanwhile, the up-regulation of seven homologues of Fatty Acid Desaturase (FAD) genes (Table S8) suggested the enhanced production of unsaturated fatty acids and the consequent elevation of membrane fluidity in *B. purpurascens*. In previous studies, over-expression of *Arabidopsis FAD7* in tobacco exhibited lesser chilling injuries compared to the wild-type [36]. Similarly, the peach fruit that was exposed to cold stress up-regulated FAD genes, delayed the degradation of phospholipids, and altered the degree of fatty acid unsaturation to mitigate the cold damage [37].

### Ca^2+^ signal under cold stress

We noted that Ca^2+^ signaling related genes exhibit significant expression changes under cold stress, suggesting that *B. purpurascens* might stimulate Ca^2+^ signaling to transfer extracellular signals to the intracellular to improve its adaptive capacity to cope with the cold environment. To our data, 8 *CNGC* homologues in *B. purpurascens* were induced in response to cold stimulus, showing that low temperature may activate CNGC channels, allowing Ca^2+^ to enter the cell. Similarly, analysis of CNGCs expression patterns in *Brassica oleracea* revealed that half of the *CNGCs* were up-regulated after incubation at 4 °C [38]. *CNGC9* was shown to regulate chilling tolerance by mediating cytoplasmic calcium elevation in rice [39]. In *Arabidopsis*, *CNGC4* was also reported to be associated with low-temperature tolerance [40]. Ca^2+^ sensors (CaMs, CMLs, CDPKs, CBLs, and CIPKs) play a role in sensing the Ca^2+^ signaling and would be induced or inhibited by abiotic stress in various plants [41]. CaMs/CMLs and CBLs are considered to be non-catalytic relay sensors, while CDPKs are acknowledged as sensory responders with catalytic activity [24]. Our data indicated that a large number of genes encoding *CAMs/CMLs, CDPKs*, and *CIPKs* significantly responded to cold stress (Fig. 3, Table S4). Among these sensor genes, three *CMLs* homologues (DN42086_c0_g1, DN9345_c0_g2, and DN9345_c0_g1) were significantly increased and highly expressed in *B. purpurascens* under cold stress, which can be considered for further validation of their functions and exploration of downstream genes. The *Arabidopsis* CML gene family comprises 50 genes, several of which were found to respond to diverse biotic and abiotic stressors. [42]. In *Medicago truncatula*, *CML5* positively regulated the expression of *CBF42* and *CBF1* to increase cold tolerance [43]. *CML10* was identified to interact with cytosolic enzymes *GSTU8* and *FBA6* to regulate cold tolerance in *Medicago sativa* [44].

### MAPK signal under cold stress

Through the analysis of transcriptome data, genes related to MAPK cascades (Fig. 4, Table S5) were induced by cold stress, which might enhance the cold tolerance of *B. purpurascens*. The MEKK1-MKK2-MPK4/MPK6 pathway is one of the well-studied MAPK cascades functioning under cold stress [45]. In *Arabidopsis*, it has been shown that cold temperature induces phosphorylation of MKK2 protein, MKK2 stimulates phosphorylation of MPK4/MPK6 protein, and MPK4/MPK6 regulates cold tolerance in plants by regulating *ICE1* and *CBF* gene expression [46]. CRLK1, a calcium/calmodulin-regulated receptor-like kinase, has been shown to interact with MEKK1 to link calcium signaling and MAPK cascades [47]. In contrast to earlier findings, the *CRLK1* homologue (DN14329_c0_g1) in *B. purpurascens* was down-regulated under cold stress (Table S4), which might be caused by negative feedback regulation in *B. purpurascens* or other unidentified factors. It is worth noting that in addition to the MEKK1-MKK2-MPK4/MPK6 pathway, the MAPK module consisting of MAP3K17/18-MKK3- MPK1/2/7/14 may also play a role in cold tolerance in *B. purpurascens*. This pathway could interact directly or indirectly with ABA signaling [48]. It has been shown that ABA induced the activation of a MAPK-like enzyme in epidermal tissue from peas [49]. Danquah et al. [50] revealed that *MAP3K17* and *MAP3K18* were regulated by the ABA core signaling module in *Arabidopsis*.

### Plant hormones signal under cold stress

Hormones generally participate in regulating plant response to cold stress [51]. Under cold stress, plants increase ABA accumulation and activate the ABA signal pathway to tolerate stress [52]. 9-cis-epoxy carotenoid dioxygenase (NCED) is one of the rate-limiting enzymes in ABA biosynthesis [53]. It has been shown that the expression level of *NCED* was up-regulated after cold stress in some plants like blueberries [32], *Pyrus ussuriensis* [54], and *Moso bamboo* [55]. Similarly, we noticed that 3 *NCED* homologues (DN41962_c0_g1, DN9413_c0_g1, DN9413 c0_g2) in *B. purpurascens* were up-regulated under cold stress (Figure S3), suggesting that low temperature might increase endogenous ABA levels. *PYL* and *PP2C* are key genes in the ABA signaling pathway, and water dissipation will decrease when their expression levels increase [56]. In the present study, *PYLs* and *PP2Cs* were up-regulated in response to cold stress. Auxin also plays an important role under cold stress in plants such as *Arabidopsis* [57], rice [58], and *Brassica napus* [59]. The Aux/IAA proteins are a large family of auxin co-receptors involved in various physiological and developmental processes [60]. In *Arabidopsis*, the expression of *IAA5* and *IAA19* is directly promoted by CBF1 and DREB2A TFs in response to abiotic stress [61]. Similarly, five *IAA* homologues were up-regulated in *B. purpurascens* under cold stress, but whether they interact with CBF requires further study. The expression patterns of ABA and auxin signaling pathway genes (Fig. 5, Table S6) in *B. purpurascens* under cold stress were similar to those in *Sonneratia caseolaris* [18] and *Malus sieversii* [62] under cold stress. DELLA activity is essential in GA signaling [30]. Achard et al. [63] observed that the accumulation of DELLA proteins induced by CBF1 played a synergistic role in cold acclimation in conjunction with the activation of COR pathways induced by CBF1. In this study, one highly expressed DELLA homologue (DN628_c0_g1) was induced, and another DELLA (DN12432_c0_g1) with a low expression level was reduced. It indicated that the GA signaling pathway might also be involved in cold tolerance of *B. purpurascens*.

### Transcription factors responding to cold stress

Numerous transcription factors from diverse families have been reported to be involved in plant cold resistance [64, 65]. To our data, most of the cold-induced transcription factors in *B. purpurascens* were consistent with maize [66] and asparagus bean [67]. Improving the regulatory capacity of a key transcription factor may alter the expression of multiple downstream functional genes and enhance plant cold resistance, like *ERF105* in *Arabidopsis* [68] and *WRKY63* in rice [69]. Among the transcription factors of *B. purpurascens*, *ERF026* homologue (DN8708_c0_g1) with high expression was the most responsive to cold stress. However, whether it plays a critical role in cold resistance needs further studies to prove. The AP2/ERF family includes numerous important transcription factors with AP2 DNA-binding domains. Among them, the CBF factor is a key regulator in response to cold stress. In *Arabidopsis*, CBF1/DREB1B, CBF2/DREB1C, and CBF3/DREB1A are rapidly induced by low temperature to enhance freezing resistance [70]. In addition to model plants, it has been reported that CBF plays a positive role in cold tolerance in birch [71], soybean [72], and barley [73]. In *Arabidopsis*, *ZAT10* and *WRKY33* act as positive regulators, whereas *MYB15* and *EIN3* act as negative regulators of the *CBF* genes. To our data, we identified one *CBF1* homologue, one *ZAT10*, one *MYB15*, and two WRKY33 in *B. purpurascens* (Figure S1), and their expression variations were consistent with previous studies. Additionally, the consistency of qRT-PCR and RNA-seq further indicated that *WRKY33* and *ZAT10* could be candidate genes for the following cold-tolerance functional studies (Figure S2).

## Conclusion

The transcriptomic analysis of *B. purpurascens* under low temperature revealed that 46 Ca^2+^ sensors related genes (two CaMs, 17 CMLs, 14 CDPKs, and 13 CIPKs) and 400 transcription factor genes (79 MYBs, 50 AP2/ERFs, 42 C2C2s, 32 WRKYs, 31 bHLHs, 27 NACs, and others) were responding to cold stress. Two MAPK cascades, five lipid metabolism pathways, and three kinds of plant hormone signal pathways (ABA, auxin, and GA) may play vital roles in enhancing the cold resistance of *B. purpurascens*. Five novel genes were suggested to be potential candidate genes involved in the cold tolerance of *B. purpurascens*. Additionally, the enormous amount of transcriptome data generated here will fill the gap in *B. purpurascens* transcriptome data.

### Electronic supplementary material

Below is the link to the electronic supplementary material.

Supplementary Material 1: **Figure S1-S3**. **Figure S1.** The expression profiles of differentially expressed transcription factors screened related to the CBF pathway. **Figure S2.** qRT-PCR performed on *WRKY33*, *ZAT10* and 8 randomly selected genes. **Figure S3.** Expression levels of genes related to ABA synthesis in *B. purpurascens* after cold stress treatment.

Supplementary Material 2: **Figure S4.** Standard curves, amplification curves and melting curves of 18S rRNA and 10 selected DEGs for qRT-PCR.

Supplementary Material 3: **Table S1-S8**. **Table S1.** Sequences of primers validated by qRT-PCR in this study. **Table S2.** Identification of differentially expressed genes. **Table S3.** Ten up-regulated and ten down-regulated unigenes with the most significant changes in expression. **Table S4.** DEGs related to Ca^2+^ signal. **Table S5.** DEGs related to MAPK signaling. **Table S6**. DEGs related to plant hormones signal. **Table S7**. Differentially expressed transcription factors under cold stress. **Table S8.** FAD (Fatty acid desaturase) related genes.

## Data Availability

The sequence data have been deposited in the NCBI under BioProject accession number PRJNA981985 (https://dataview.ncbi.nlm.nih.gov/object/PRJNA981985).
