# Supplementary material for: Transcriptome profiling of Bergenia purpurascens under cold stress
Source: BMC Genomics. 2023 Dec 7;24:754. doi: 10.1186/s12864-023-09850-z (PMC10702111; doi:10.1186/s12864-023-09850-z)
Supplement: Supplementary file 1 — Supplementary Material 1: Figure S1-S3. Figure S1. The expression profiles of differentially expressed transcription factors screened related to the CBF pathway. Figure S2. qRT-PCR performed on WRKY33, ZAT10 and 8 randomly selected genes. Figure S3. Expression levels of genes related to ABA synthesis in B. purpurascens after cold stress treatment. [file 12864_2023_9850_MOESM1_ESM.doc]

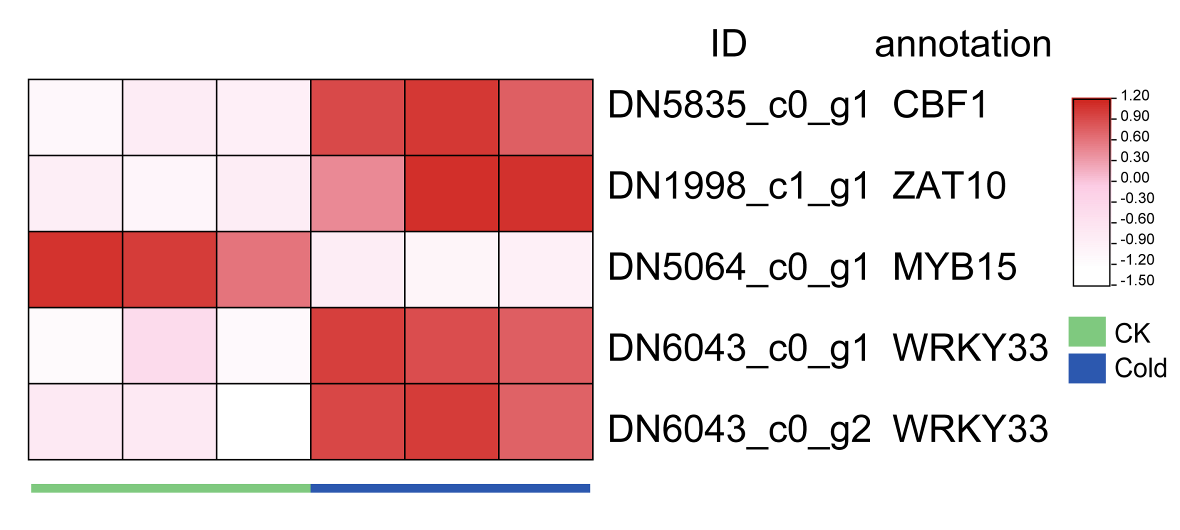


**Figure S1** The expression profiles of differentially expressed transcription factors screened related to the CBF pathway.





**Figure S2** qRT-PCR performed on *WRKY33*, *ZAT10* and 8 randomly selected genes. Relative expression levels of genes examined were calculated and expressed as 2^‑ΔΔCT^. RNA-seq value was based on fold change of up-regulated or down-regulated DEGs. Error bars indicate SD. The significance test was determined using Student’s t test (*P < 0.05, **P < 0.01, ***P < 0.001, ****P < 0.0001).


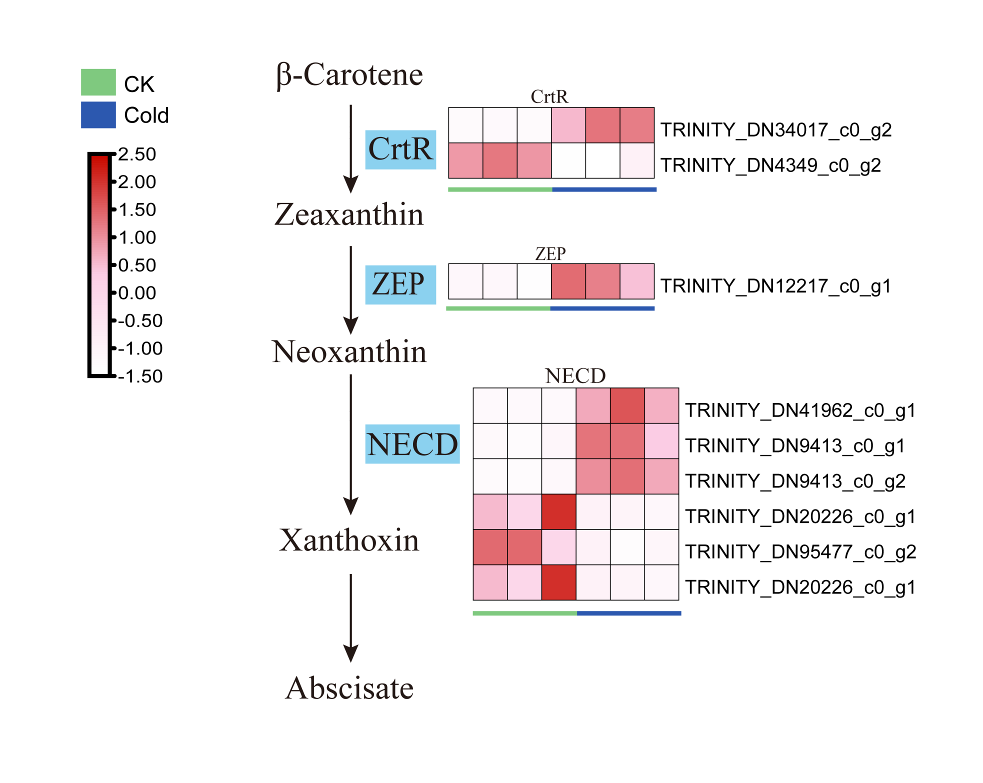


**Figure S3** Expression levels of genes related to ABA synthesis in *B. purpurascens* after cold stress treatment. Crtz (beta-carotene 3-hydroxylase), ZEP (zeaxanthin epoxidase) and NCED (9-cis-epoxycarotenoid dioxygenase) are ABA biosynthesis enzymes. Neoxanthin catalyzed by 9-cis-epoxycarotenoid dioxygenases (NCEDs) to induce flavotoxins is one of the rate-limiting steps in ABA biosynthesis.
