# Supplementary material for: Transcriptome profiling of Bergenia purpurascens under cold stress
Source: BMC Genomics. 2023 Dec 7;24:754. doi: 10.1186/s12864-023-09850-z (PMC10702111; doi:10.1186/s12864-023-09850-z)
Supplement: Supplementary file 2 — Supplementary Material 2: Figure S4. Standard curves, amplification curves and melting curves of 18S rRNA and 10 selected DEGs for qRT-PCR. [file 12864_2023_9850_MOESM2_ESM.doc]

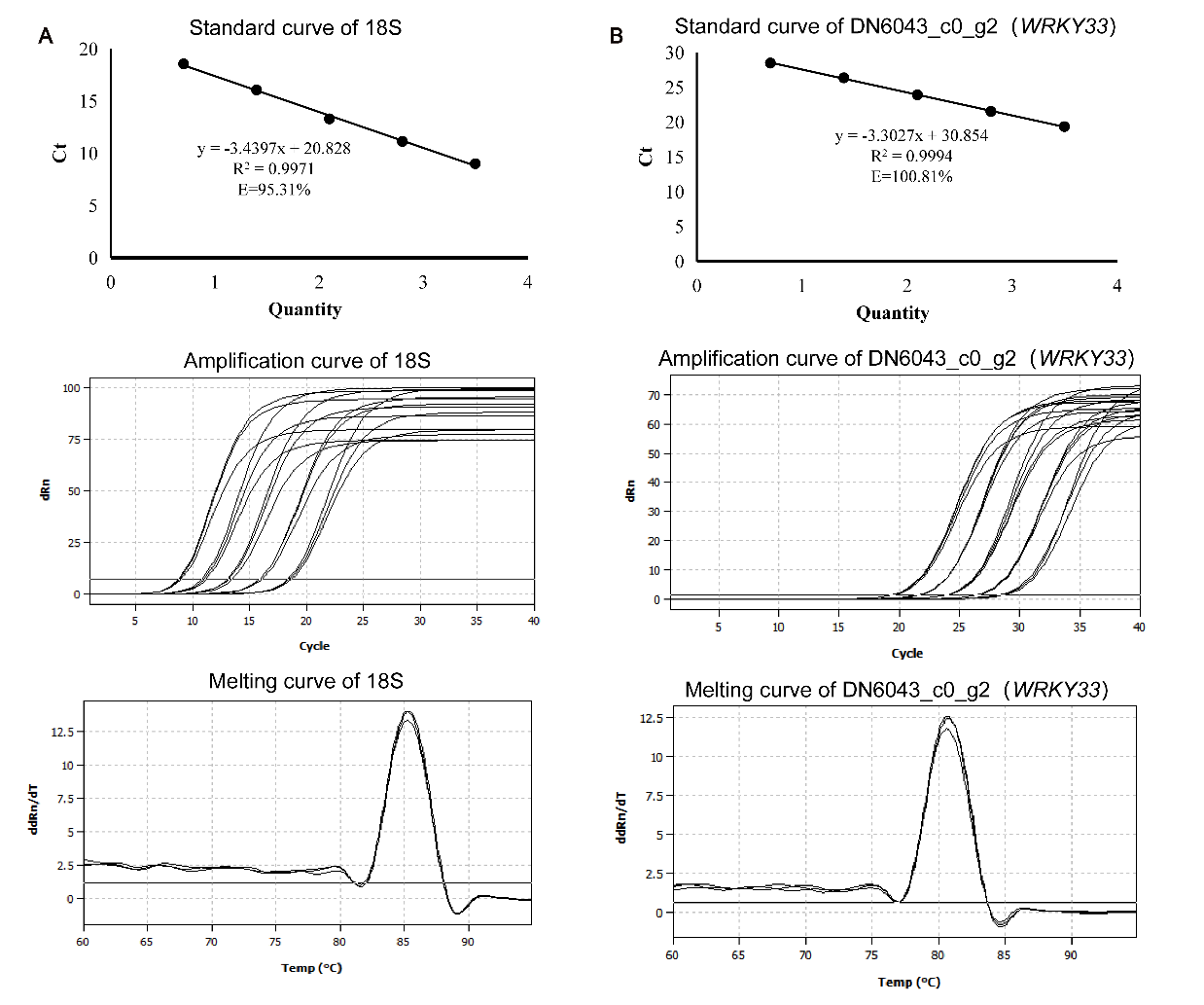


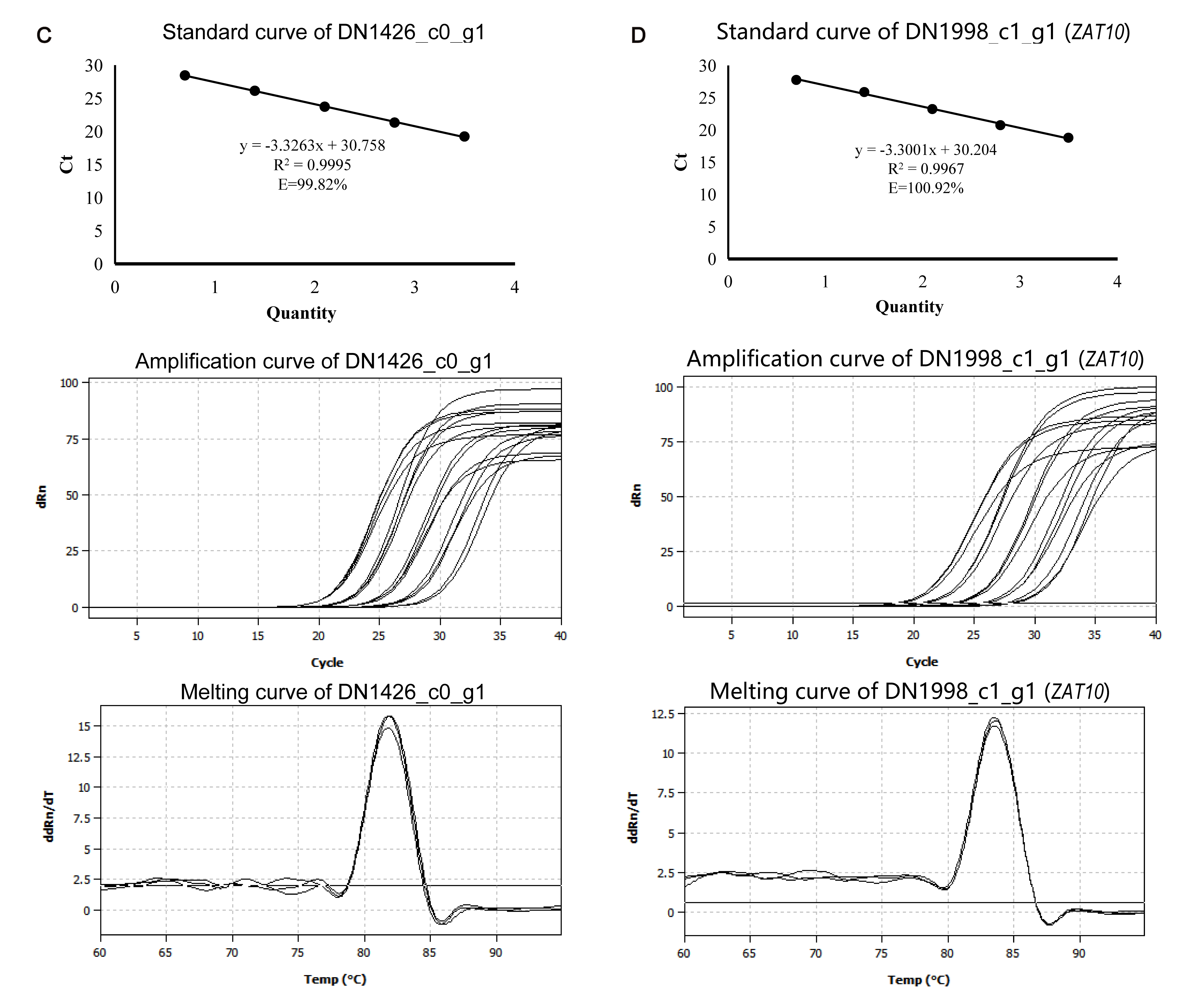


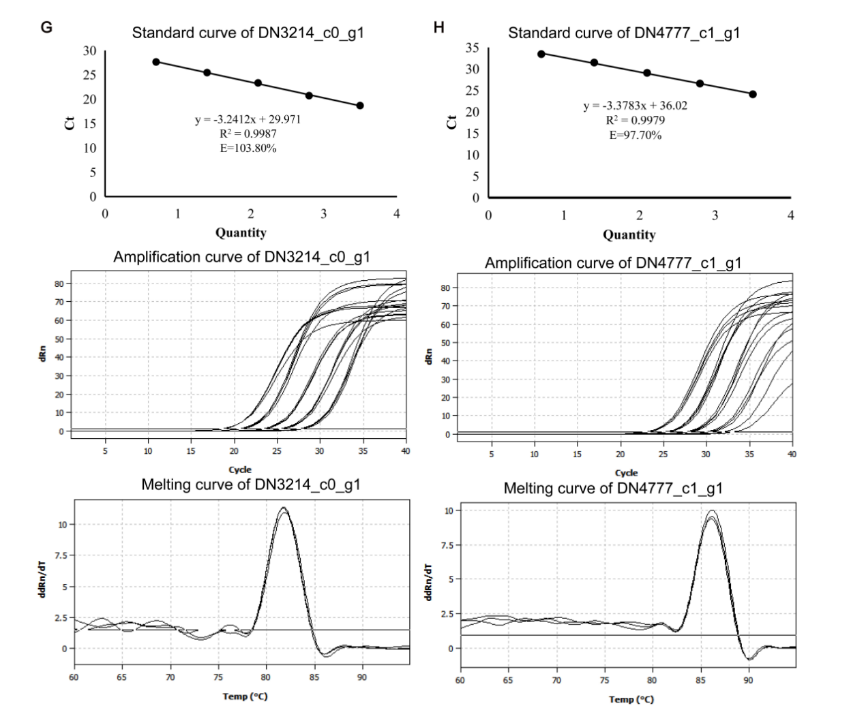

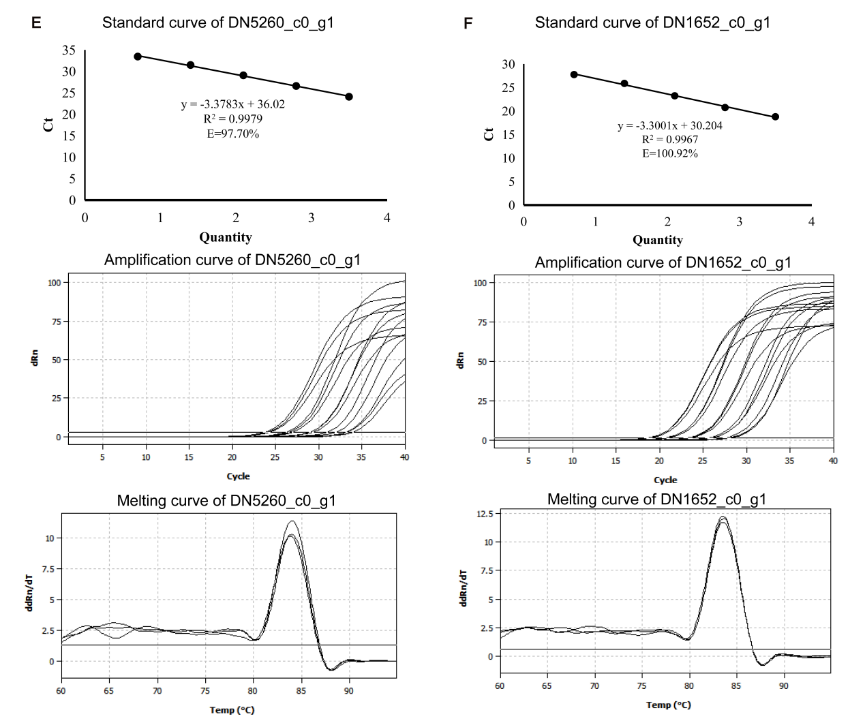


**
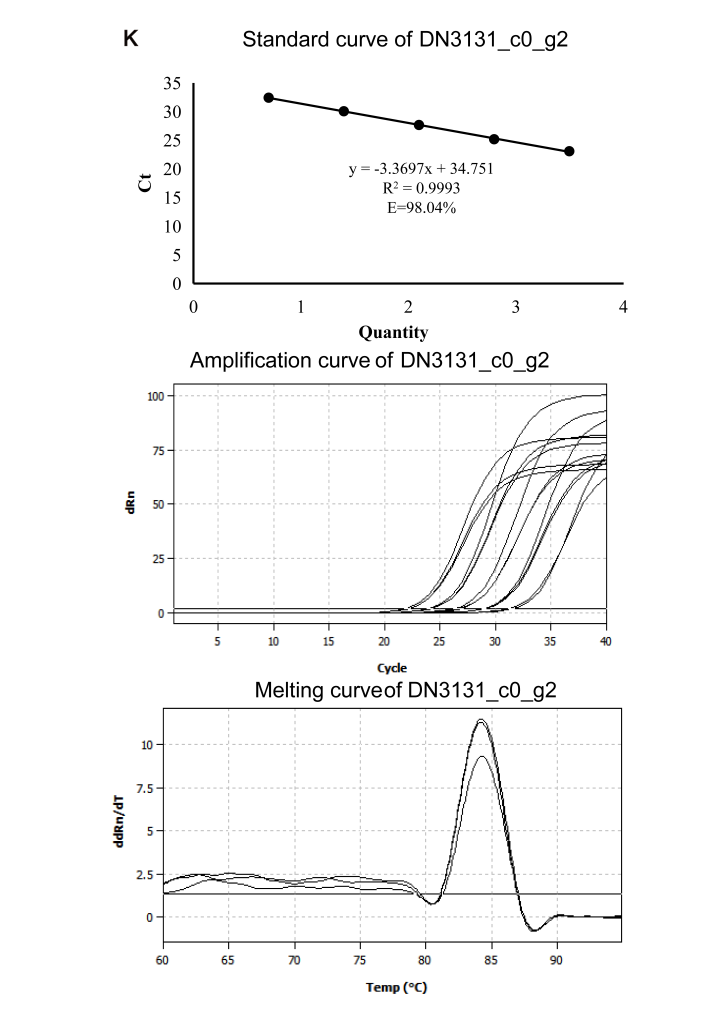
**
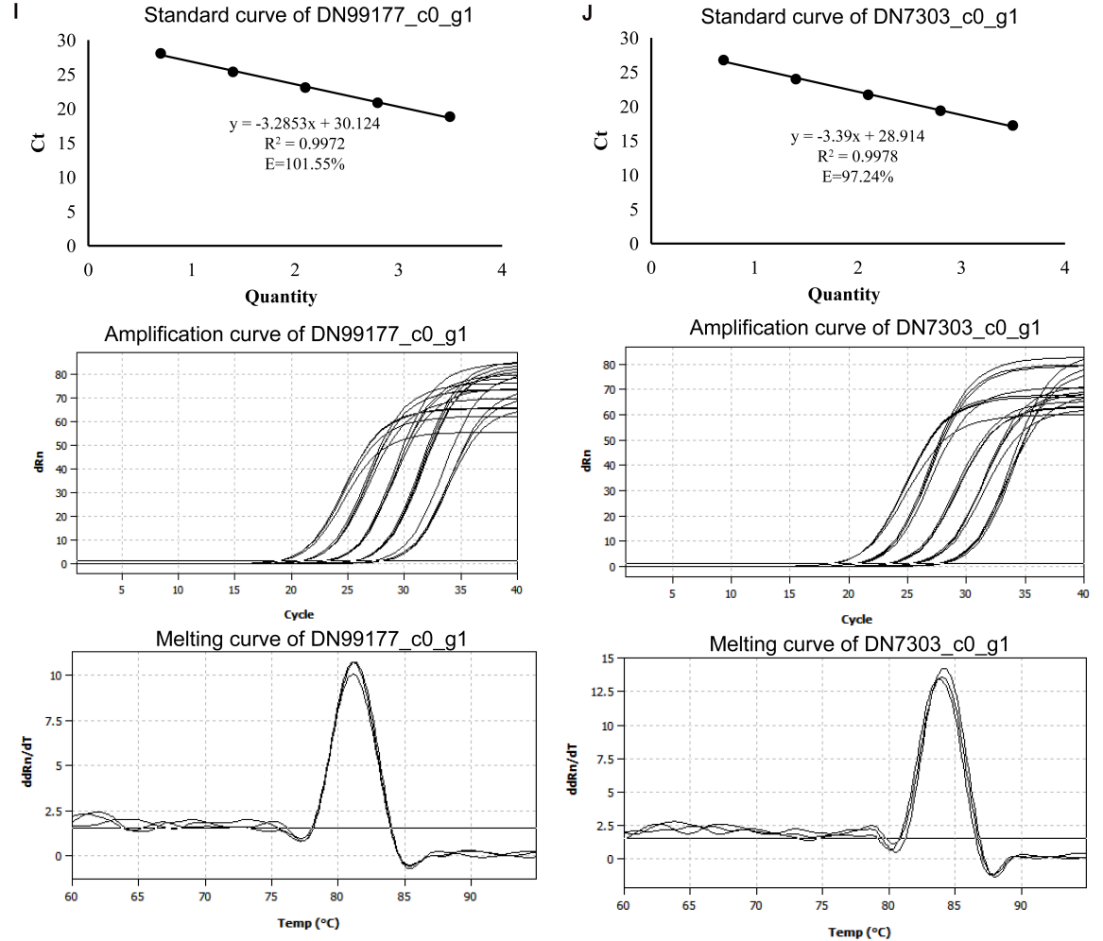


**Figure S4.** Standard curves, amplification curves and melting curves of 18S rRNA and 10 selected DEGs for qRT-PCR. (A) 18S rRNA, (B) DN6043_c0_g2 (*WRKY33*), (C) DN1426_c0_g1, (D) DN1998_c1_g1 (*ZAT10*), (E) DN5260_c0_g1, (F) DN1652_c0_g1, (G) DN3214_c0_g1, (H) DN4777_c1_g1, (I) DN99177_c0_g1, (J) DN7303_c0_g1, (K) DN3131_c0_g2.
